# Author Correction: Control of replication and gene expression by ADP-ribosylation of DNA in *Mycobacterium tuberculosis*

**DOI:** 10.1038/s44318-025-00491-4

**Published:** 2025-06-25

**Authors:** Rachel E Butler, Marion Schuller, Ritu Jaiswal, Jayanta Mukhopadhyay, Jim Barber, Suzie Hingley-Wilson, Emily Wasson, Alexessander Couto Alves, Ivan Ahel, Graham R Stewart

**Affiliations:** 1 Section of Bacteriology, School of Biosciences, University of Surrey, Guildford, Surrey GU2 7XH UK; 2 Sir William Dunn School of Pathology, University of Oxford, Oxford, OX1 3RE UK; 3 Department of Chemical Science, Bose Institute, EN80 Sector V, Salt Lake, Kolkata, West Bengal 700091 India; 4 School of Human Development and Health, University of Southampton, Southampton, SO16 6YD UK

## Abstract

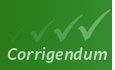

**Correction to:** *The EMBO Journal* (2025) 44:3468–3491. 10.1038/s44318-025-00451-y | Published online 8 May 2025

**The 8th** **Author’s name and affiliation are corrected**

The 8th Author’s name is corrected from Alex Couto Alves

To: (Change in bold).

**Alexessander** Couto Alves

The 8th Author’s affiliation is corrected from

^1^Section of Bacteriology, School of Biosciences, University of Surrey, Guildford, Surrey GU2 7XH, United Kingdom

To: (Change in bold).

^**4**^ **School of Human Development and Health, University of Southampton, Southampton SO16 6YD, United Kingdom**

