# Supplementary material for: Control of replication and gene expression by ADP-ribosylation of DNA in Mycobacterium tuberculosis
Source: EMBO J. 2025 May 8;44(12):3468–91. doi: 10.1038/s44318-025-00451-y (PMC12170906; doi:10.1038/s44318-025-00451-y)
Supplement: Supplementary file 1 — Table EV1 [file 44318_2025_451_MOESM1_ESM.docx]

Table EV1. Bacterial Strains and Plasmids

| **Strain or plasmid-ID** | **Description** | **Antibiotic resistance** | **Source** |
| --- | --- | --- | --- |
| *E. coli* BL21(DE3) pLysS Singles™ | F^-^ *ompT hsdS*_B_(r_B_^-^ m_B_^-^) *gal dcm* (DE3) pLysS (Cam^R^) | Cam^R^ | Novagen |
| *E. coli* DH5α-macro | DH5α with integrated Thermus aquaticus DarG macrodomain at  P21 site |  | Schuller et al 2021 |
| *M. bovis* BCG | BCG / Pasteur 1173P2 |  | Lab stocks |
| *M. bovis* BCG *darG* sgRNA | *BCG carrying pRH2502 and pRH2521-darG-sgRNA* (pRH5982) | Kan^R^  Hyg^R^ | Schuller et al 2021 |
| *M. bovis* BCG *darG2* sgRNA | *BCG carrying darG-knockdown plasmid pIJR965-DarG* | Kan^R^ | This study |
| *M. tuberculosis* GC1237 | Caminero *et al*, 2001. |  | Lab Stocks |
| *M. tuberculosis ΔdarTdarG* | *M. tuberculosis* GC1237 *ΔdarTdarG* | Hyg^R^ | Schuller et al 2021 |
| *pETDuet rpoB-rpoC* | *Expression plasmid* | Amp^R^ | Banerjee et al 2014 |
| *pAcYc Duet-rpoA-sigA* | *Expression plasmid* | Chl^R^ | Banerjee et al 2014 |
| pAcYc Duet-*rpoA-rpoz* | *Expression plasmid* | Chl^R^ | Banerjee et al 2014 |
| pBAD33_Mtb_darT | pBAD33 carrying *M. tuberculosis darT* | Cam^R^ | Schuller et al 2021 |
| Zur_pET151/D-TOPO | N-terminal His-6 tagged expression plasmid, with TEV cleavage site | Amp^R^ | This study |
