# Supplementary material for: Control of replication and gene expression by ADP-ribosylation of DNA in Mycobacterium tuberculosis
Source: EMBO J. 2025 May 8;44(12):3468–91. doi: 10.1038/s44318-025-00451-y (PMC12170906; doi:10.1038/s44318-025-00451-y)
Supplement: Supplementary file 6 — Movie EV1 [file 44318_2025_451_MOESM6_ESM.zip › Expanded View Movie EV1/Expanded View Movie EV1 Figure Legend.docx]

**Expanded View Movie EV1**. **Replication of BCG carrying an empty CRISPRi vector (control)**. BCG were pre-treated for 48h with 200ng/ml ATC, then loaded into an ONIX microfluidic device and imaged every hour on a confocal microscope with continued ATC treatment.
