# Supplementary material for: Control of replication and gene expression by ADP-ribosylation of DNA in Mycobacterium tuberculosis
Source: EMBO J. 2025 May 8;44(12):3468–91. doi: 10.1038/s44318-025-00451-y (PMC12170906; doi:10.1038/s44318-025-00451-y)
Supplement: Supplementary file 8 — Movie EV3 [file 44318_2025_451_MOESM8_ESM.zip › Expanded View Movie EV3/Expanded View Movie EV3 Figure Legend.docx]

**Expanded View Movie EV3. Resumption of replication after release of *darG*-knockdown.** BCG *darG* sgDNA were minimally inhibited with ATC to knockdown DarG expression in a microtitre plate for 7 days, loaded into an ONIX microfluidic device, and imaged every hour on a confocal microscope. After 12 hours, ATC was washed out of the device to re-establish DarG expression.
