# Supplementary figures and images for: Control of replication and gene expression by ADP-ribosylation of DNA in Mycobacterium tuberculosis
Source: EMBO J. 2025 May 8;44(12):3468–91. doi: 10.1038/s44318-025-00451-y (PMC12170906; doi:10.1038/s44318-025-00451-y)

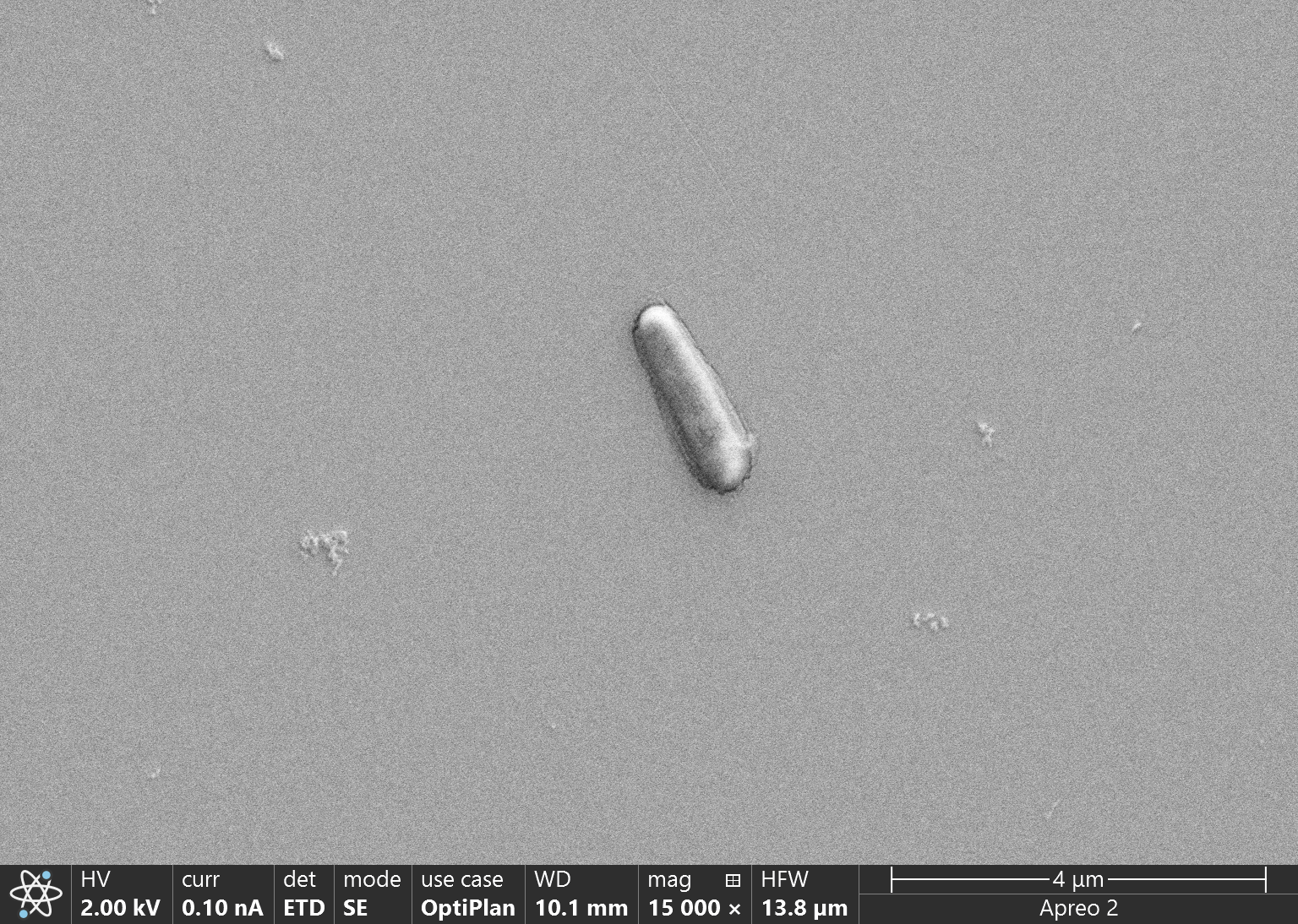

Supplement: Supplementary file 9 — Source data Fig. 1 [file 44318_2025_451_MOESM9_ESM.zip › Figure 1/1B/Images/S1_2.00kV_0.10nA_15000×_ETD_114.tif]

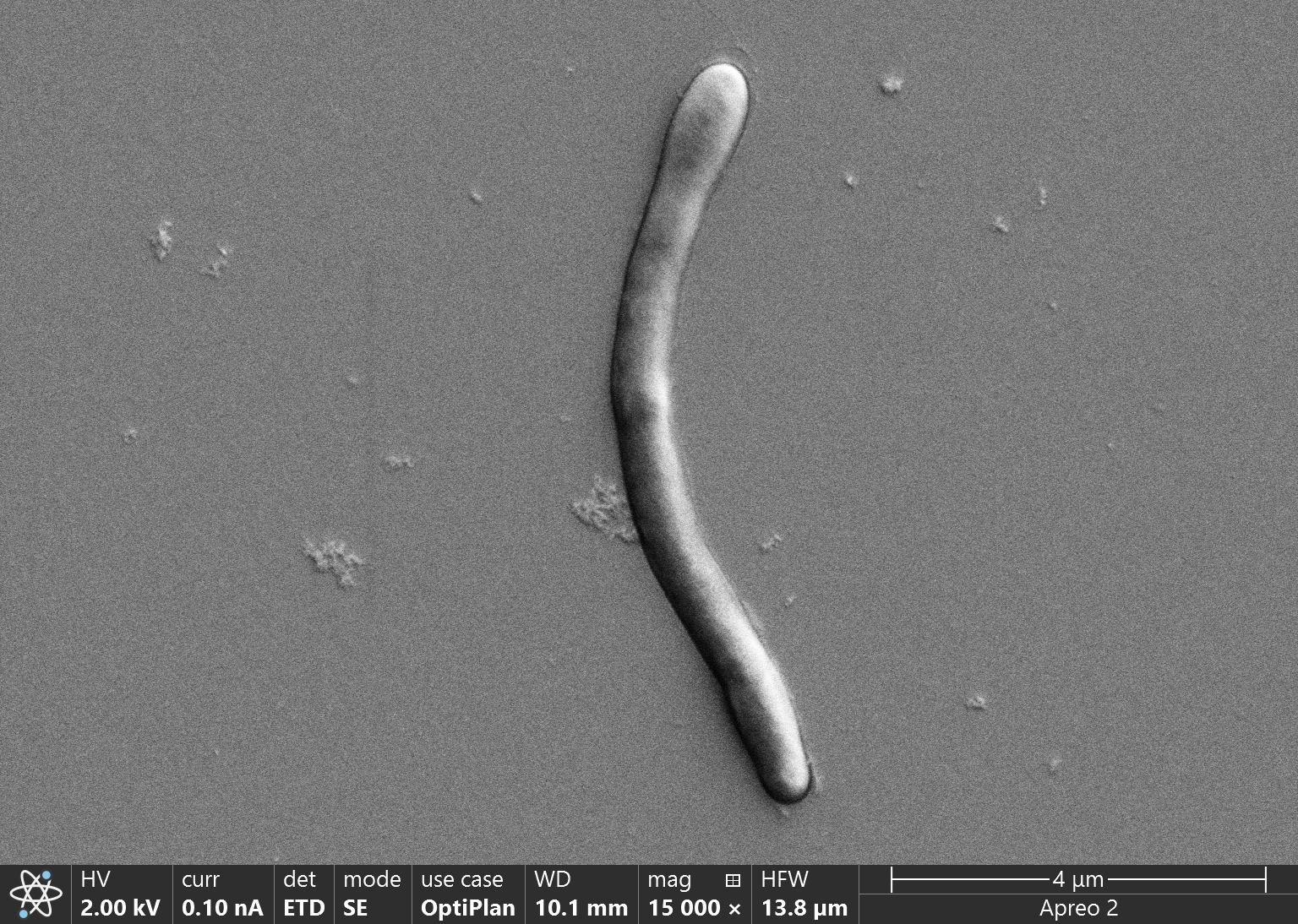

Supplement: Supplementary file 9 — Source data Fig. 1 [file 44318_2025_451_MOESM9_ESM.zip › Figure 1/1B/Images/S2_2.00kV_0.10nA_15000×_ETD_205.tif]

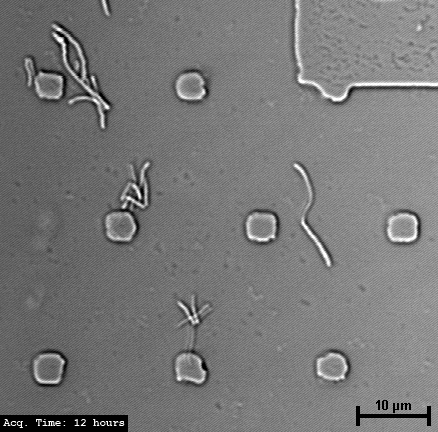

Supplement: Supplementary file 9 — Source data Fig. 1 [file 44318_2025_451_MOESM9_ESM.zip › Figure 1/1D/TIFFS/T0.tif]

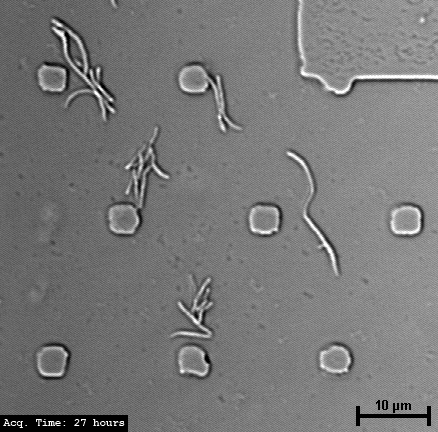

Supplement: Supplementary file 9 — Source data Fig. 1 [file 44318_2025_451_MOESM9_ESM.zip › Figure 1/1D/TIFFS/T15.tif]

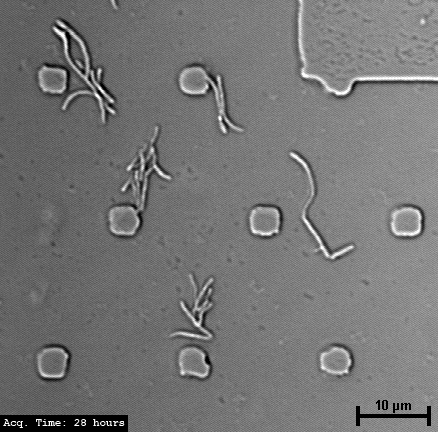

Supplement: Supplementary file 9 — Source data Fig. 1 [file 44318_2025_451_MOESM9_ESM.zip › Figure 1/1D/TIFFS/T16.tif]

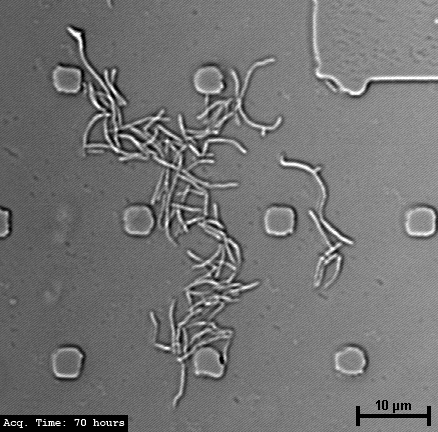

Supplement: Supplementary file 9 — Source data Fig. 1 [file 44318_2025_451_MOESM9_ESM.zip › Figure 1/1D/TIFFS/T56.tif]

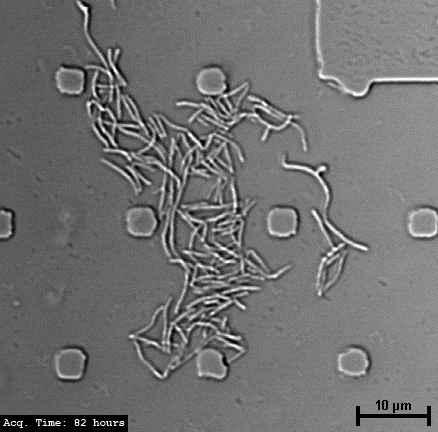

Supplement: Supplementary file 9 — Source data Fig. 1 [file 44318_2025_451_MOESM9_ESM.zip › Figure 1/1D/TIFFS/T70.tif]

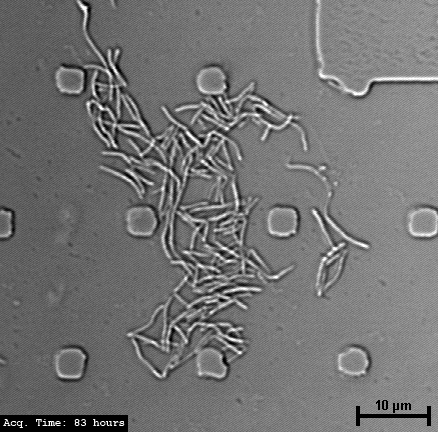

Supplement: Supplementary file 9 — Source data Fig. 1 [file 44318_2025_451_MOESM9_ESM.zip › Figure 1/1D/TIFFS/T71.tif]

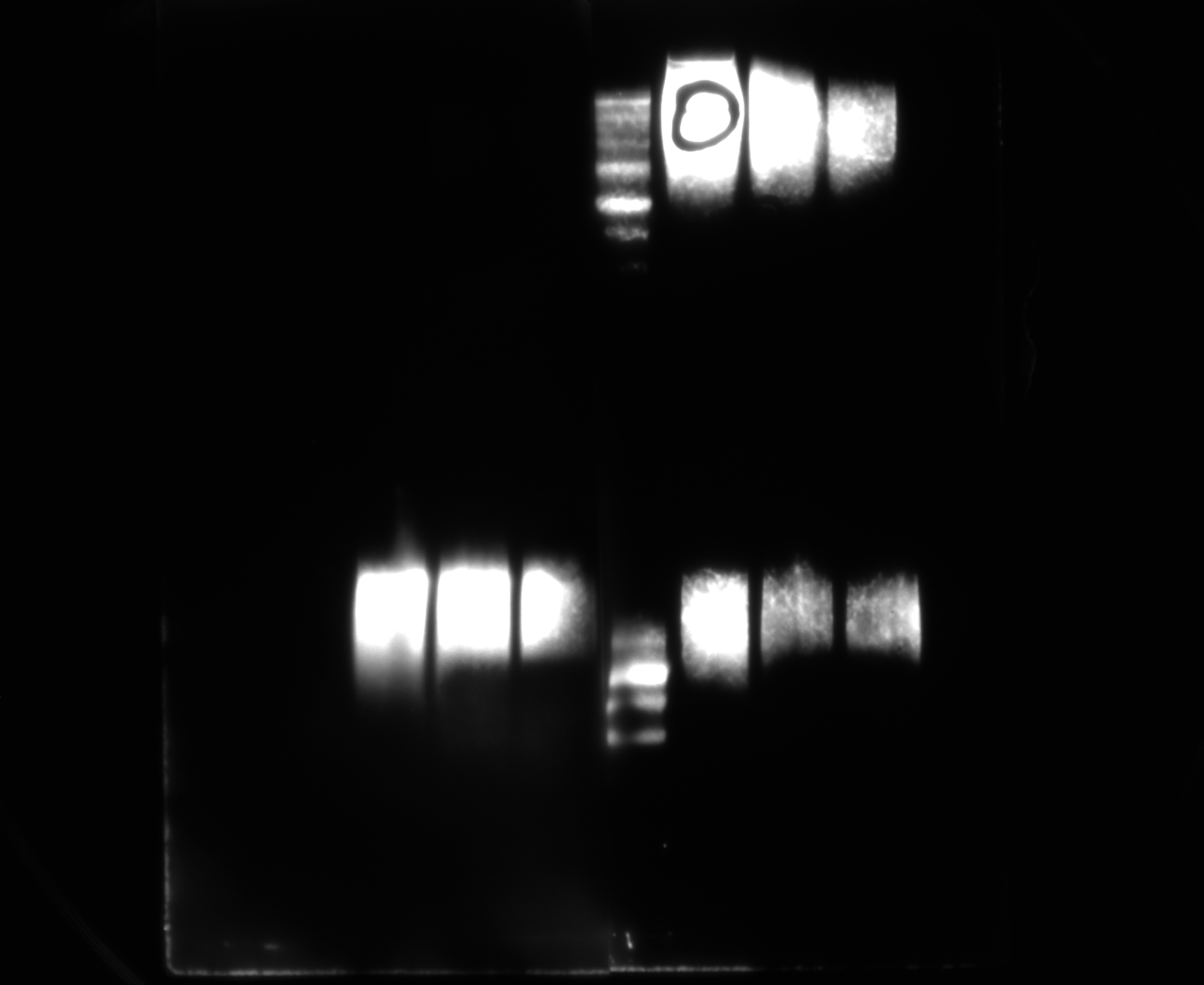

Supplement: Supplementary file 10 — Source data Fig. 2 [file 44318_2025_451_MOESM10_ESM.zip › Figure 2/2A/Antibody.Tif]

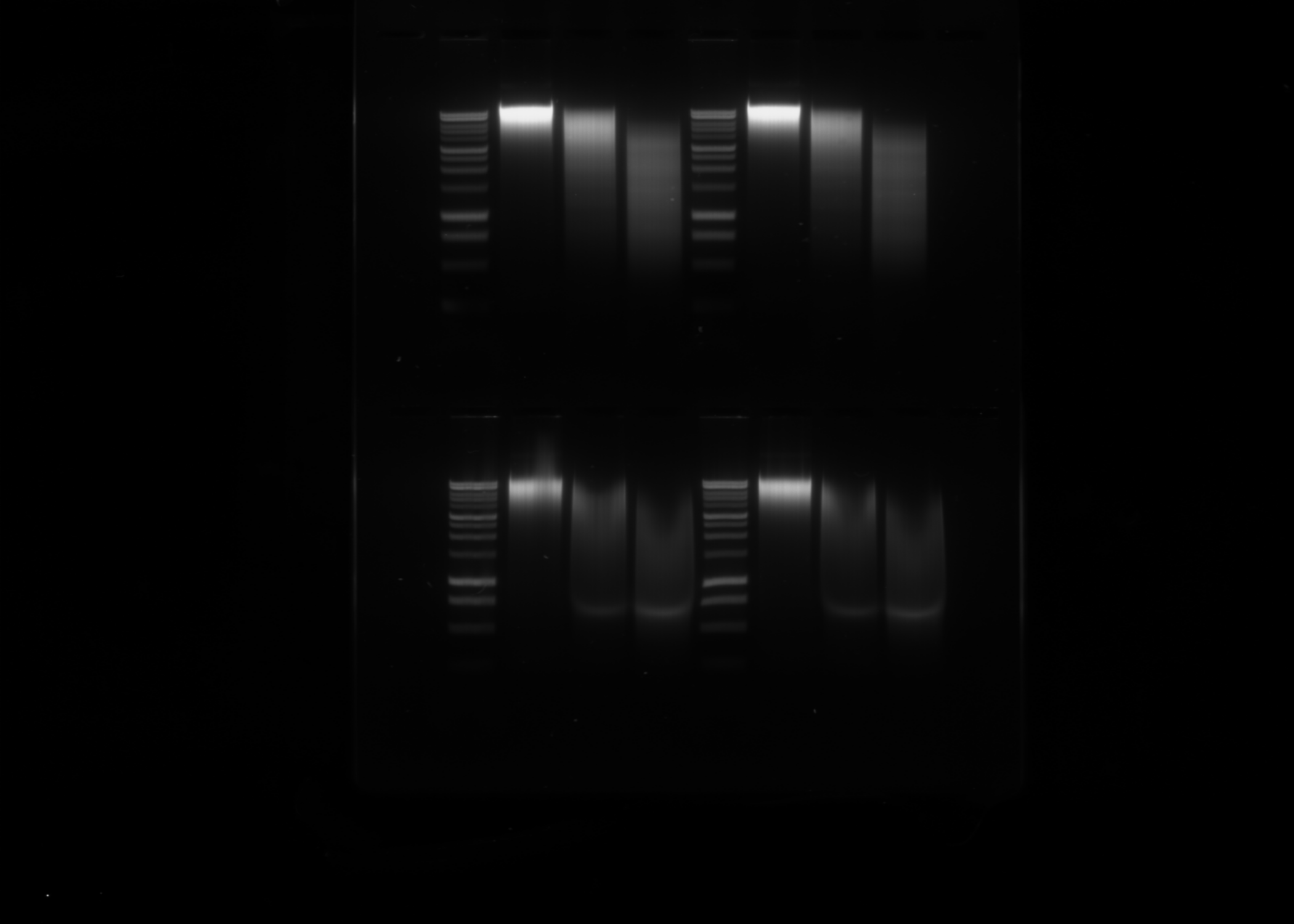

Supplement: Supplementary file 10 — Source data Fig. 2 [file 44318_2025_451_MOESM10_ESM.zip › Figure 2/2A/DNA.tif]

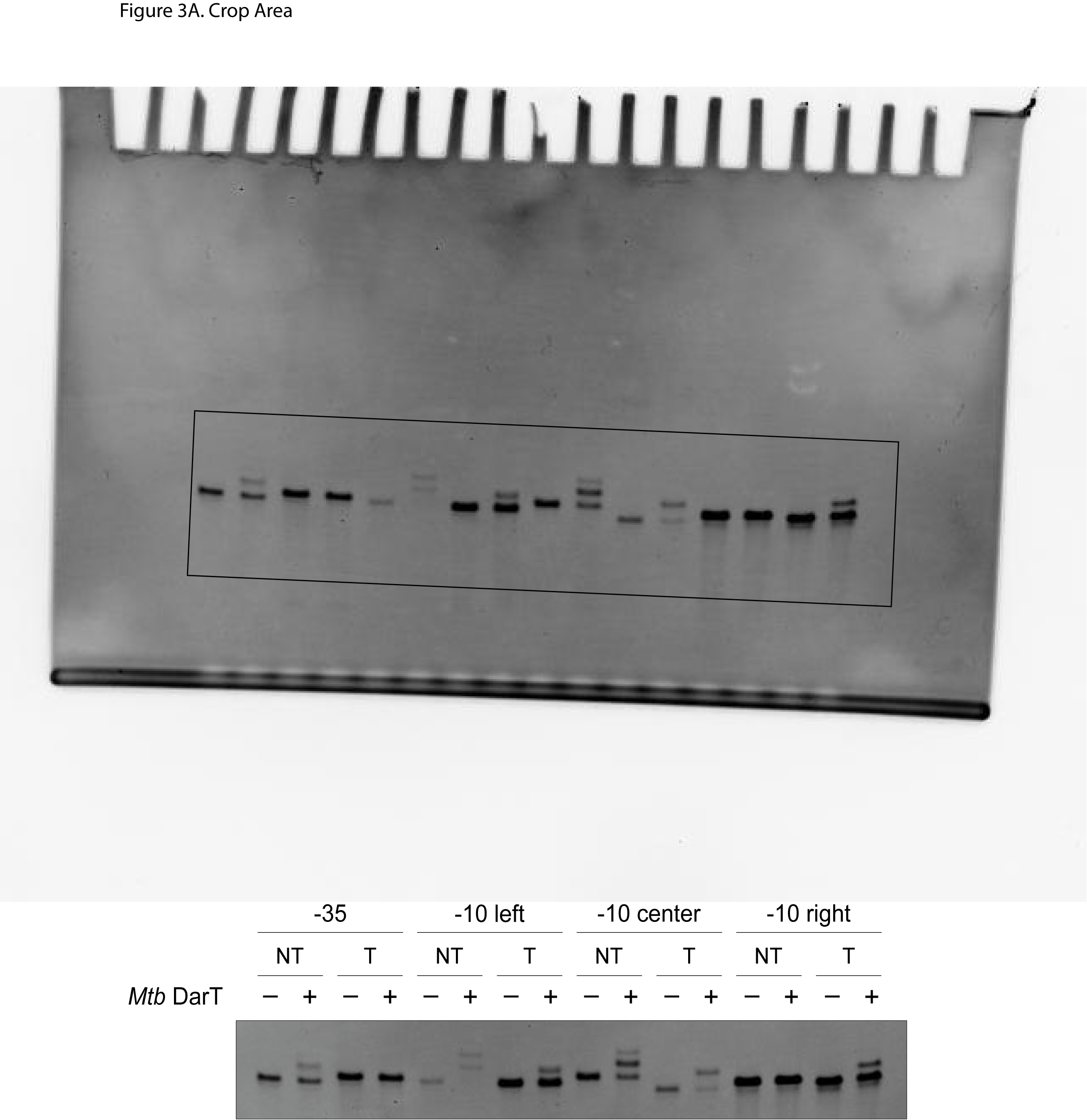

Supplement: Supplementary file 11 — Source data Fig. 3 [file 44318_2025_451_MOESM11_ESM.zip › Figure 3/Fig 3A.tif]

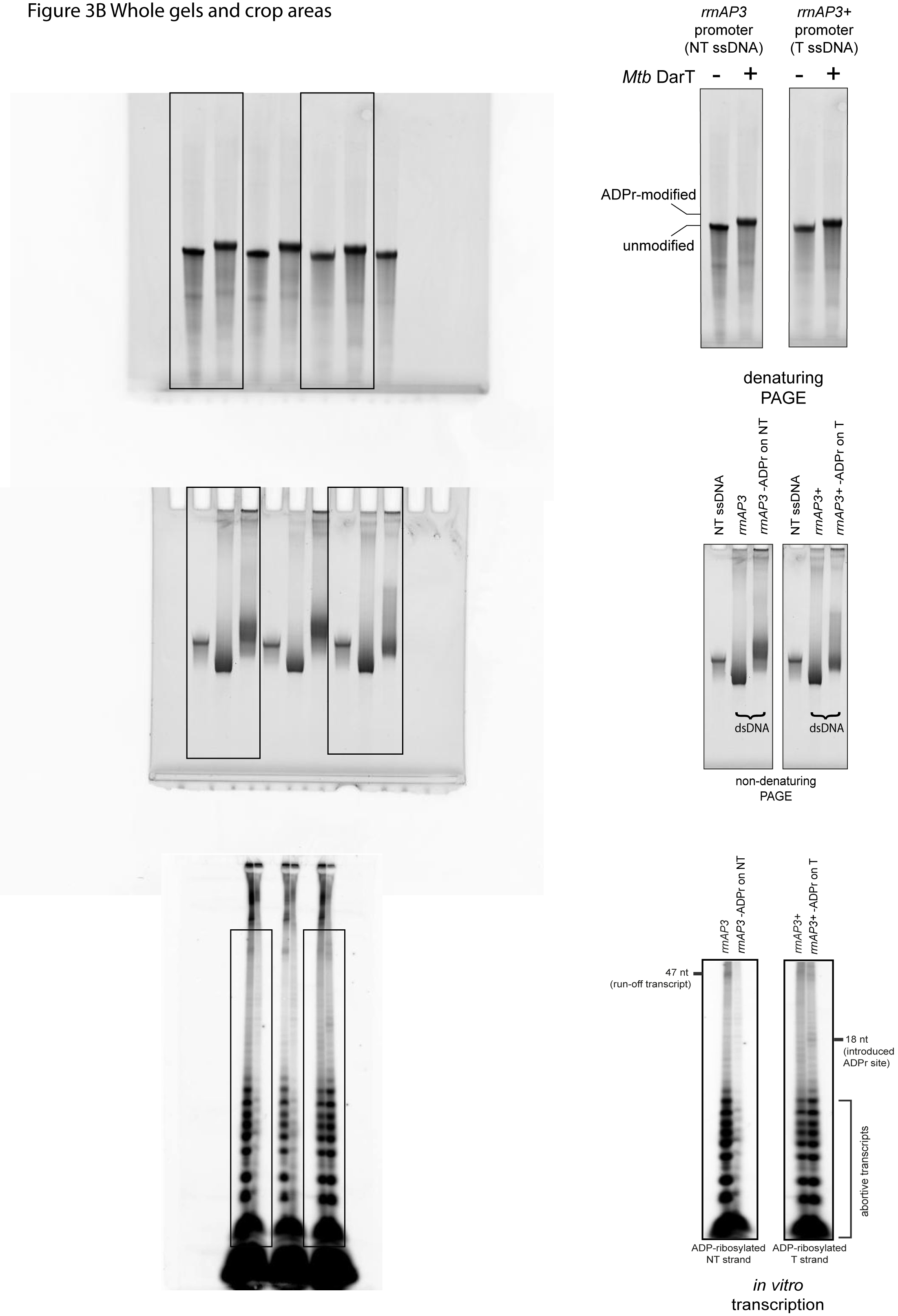

Supplement: Supplementary file 11 — Source data Fig. 3 [file 44318_2025_451_MOESM11_ESM.zip › Figure 3/Figure 3B Source Data.tif]

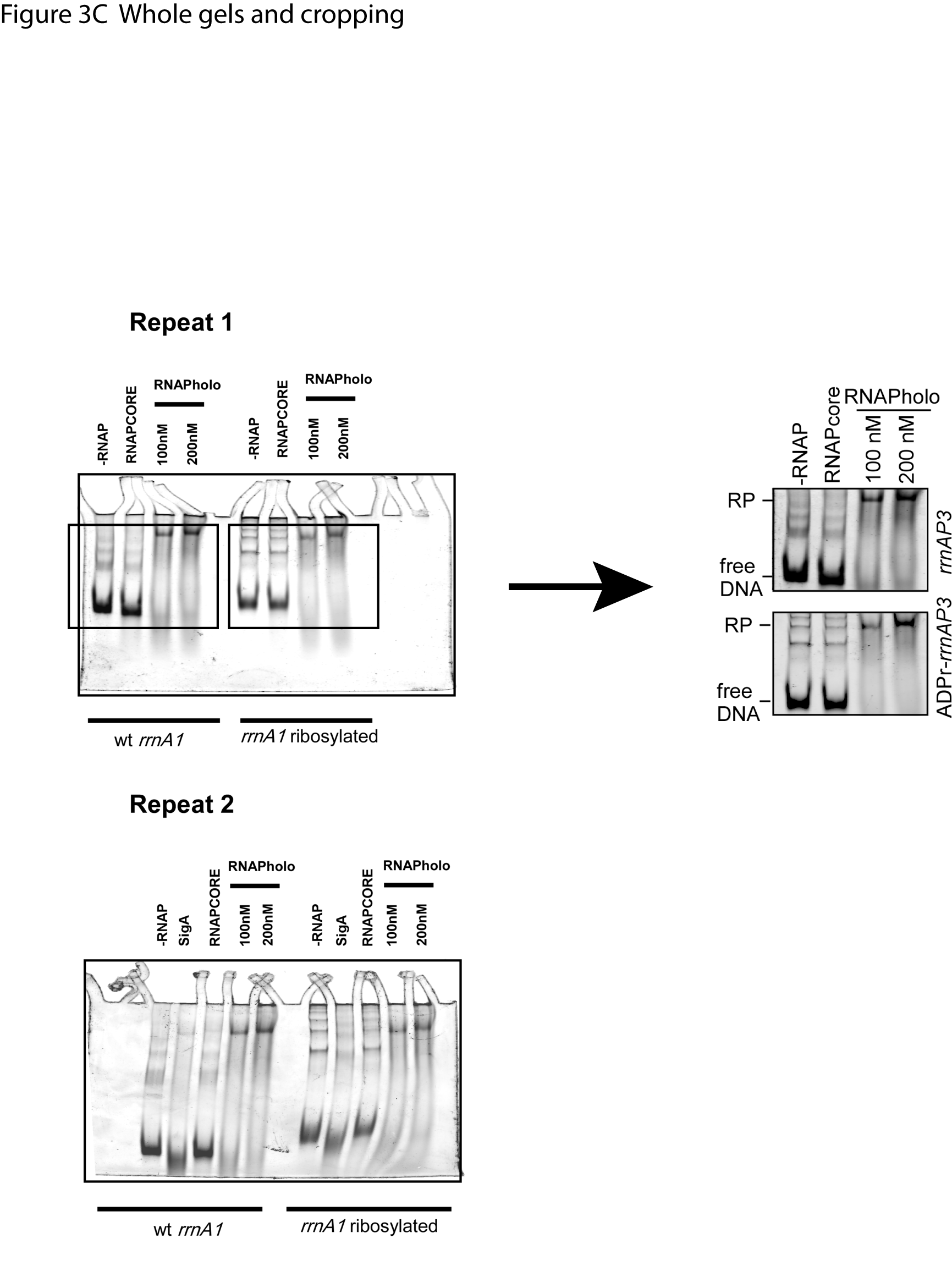

Supplement: Supplementary file 11 — Source data Fig. 3 [file 44318_2025_451_MOESM11_ESM.zip › Figure 3/Figure 3C Source Data.tif]

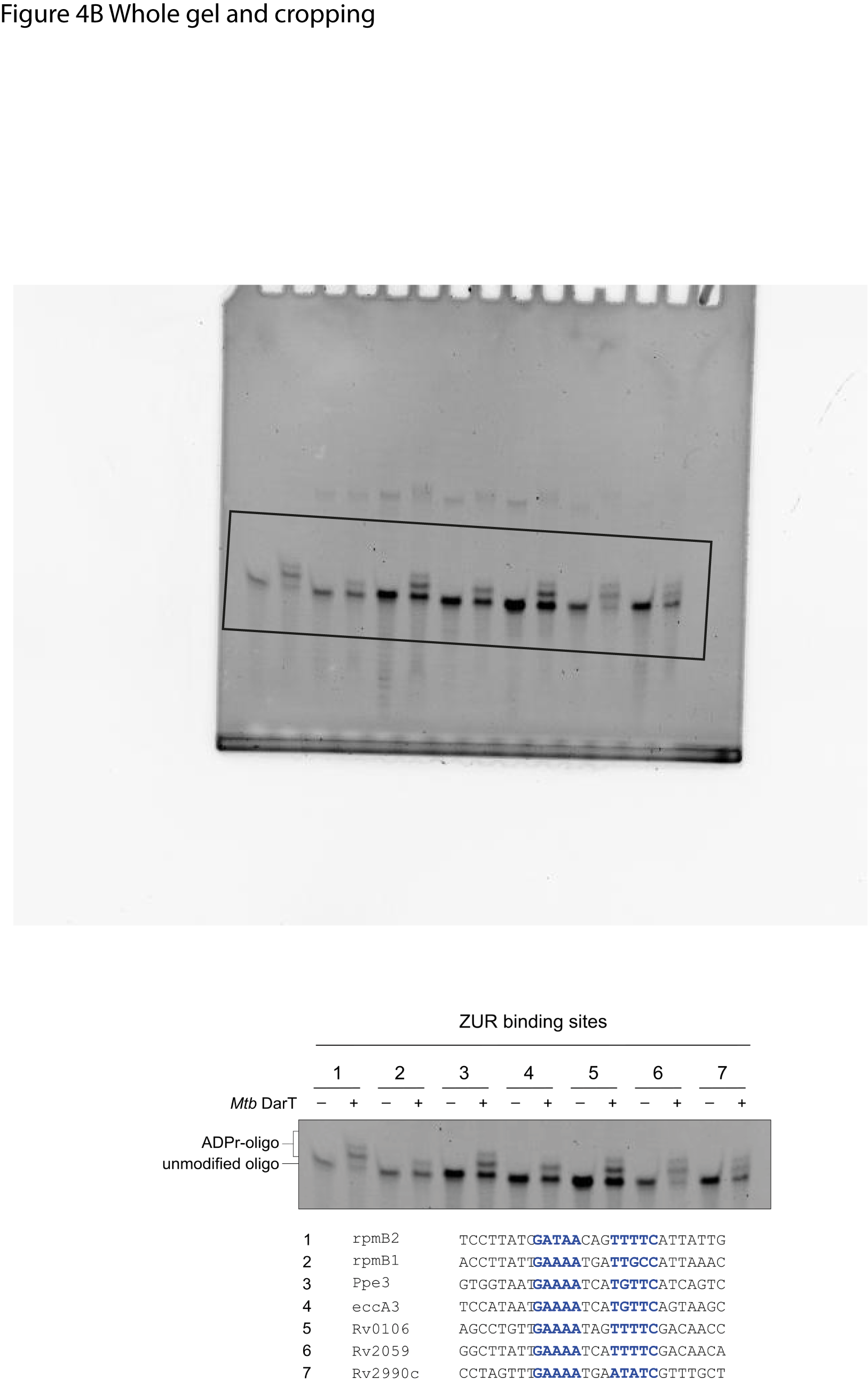

Supplement: Supplementary file 12 — Source data Fig. 4 [file 44318_2025_451_MOESM12_ESM.zip › Figure 4/Figure 4B Source data.tif]

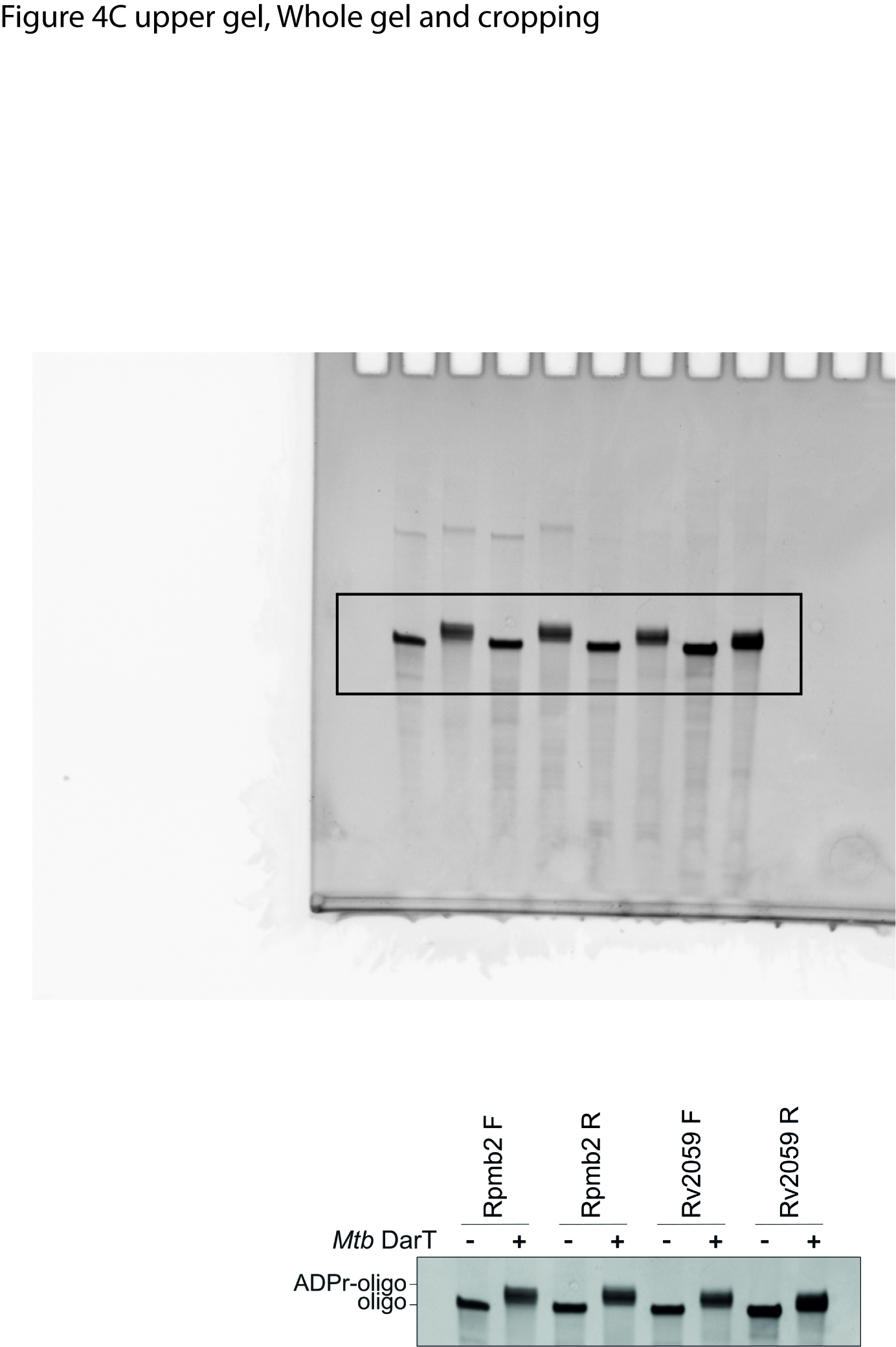

Supplement: Supplementary file 12 — Source data Fig. 4 [file 44318_2025_451_MOESM12_ESM.zip › Figure 4/4C/Figure 4C upper gel cropping.tif]

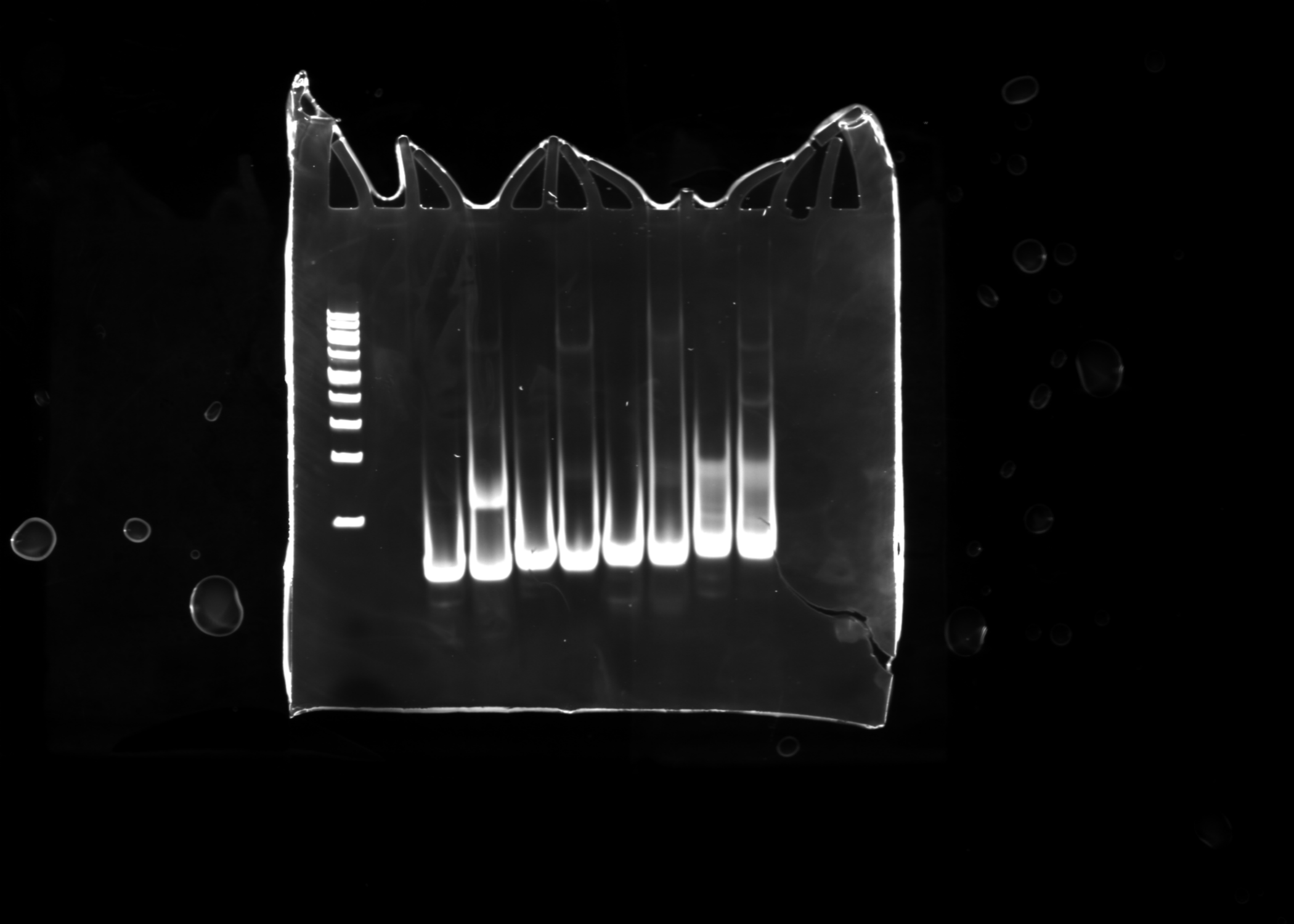

Supplement: Supplementary file 12 — Source data Fig. 4 [file 44318_2025_451_MOESM12_ESM.zip › Figure 4/4C/rpmB2.tif]

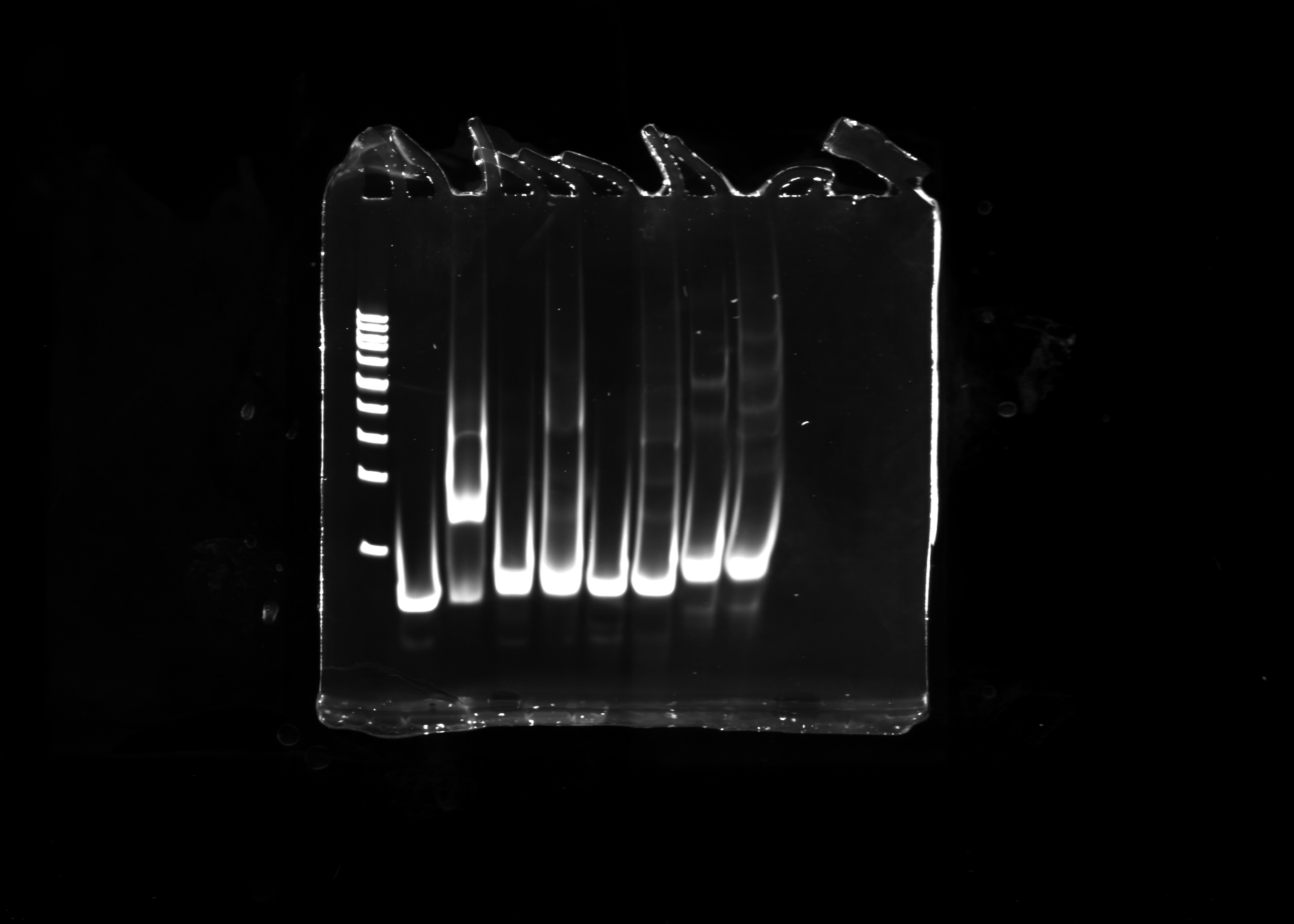

Supplement: Supplementary file 12 — Source data Fig. 4 [file 44318_2025_451_MOESM12_ESM.zip › Figure 4/4C/Rv2059.tif]
