# Supplementary material for: Control of replication and gene expression by ADP-ribosylation of DNA in Mycobacterium tuberculosis
Source: EMBO J. 2025 May 8;44(12):3468–91. doi: 10.1038/s44318-025-00451-y (PMC12170906; doi:10.1038/s44318-025-00451-y)
Supplement: Supplementary file 10 — Source data Fig. 2 [file 44318_2025_451_MOESM10_ESM.zip › Figure 2/2A/2A DNA cropping.pdf]

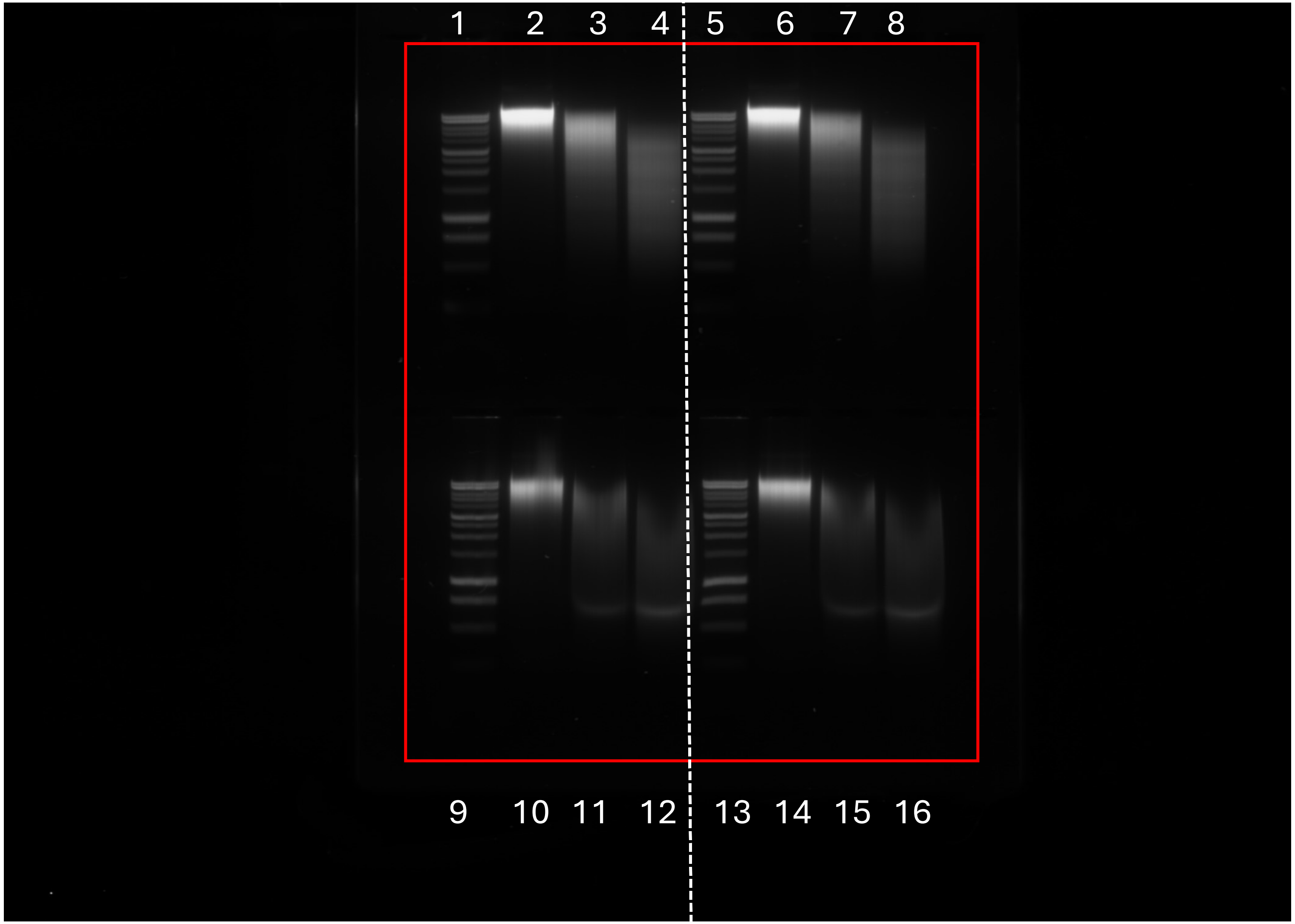

1 1kb ladder
2 Uncut gDNA (0ng/ml ATC 48h)
3 msel digested gDNA (0ng/ml ATC 48h)
4 salI digested gDNA (0ng/ml ATC 48h)

5 1kb ladder
6 Uncut gDNA (0ng/ml ATC 48h)
7 msel digested gDNA (0ng/ml ATC 48h)
8 salI digested gDNA (0ng/ml ATC 48h

9 1kb ladder
10 Uncut gDNA (200ng/ml ATC 48h)
11 msel digested gDNA (200ng/ml ATC 48h)
12 salI digested gDNA (200ng/ml ATC 48h

13 1kb ladder
14 Uncut gDNA (200ng/ml ATC 48h)
15 msel digested gDNA (200ng/ml ATC 48h)
16 salI digested gDNA (200ng/ml ATC 48h

Gel cut and transferred for anti-ADPr blot

Gel cut and transferred for anti-dsDNA blot
