# Supplementary material for: Control of replication and gene expression by ADP-ribosylation of DNA in Mycobacterium tuberculosis
Source: EMBO J. 2025 May 8;44(12):3468–91. doi: 10.1038/s44318-025-00451-y (PMC12170906; doi:10.1038/s44318-025-00451-y)
Supplement: Supplementary file 12 — Source data Fig. 4 [file 44318_2025_451_MOESM12_ESM.zip › Figure 4/4C/rpmB2 crop area.pdf]

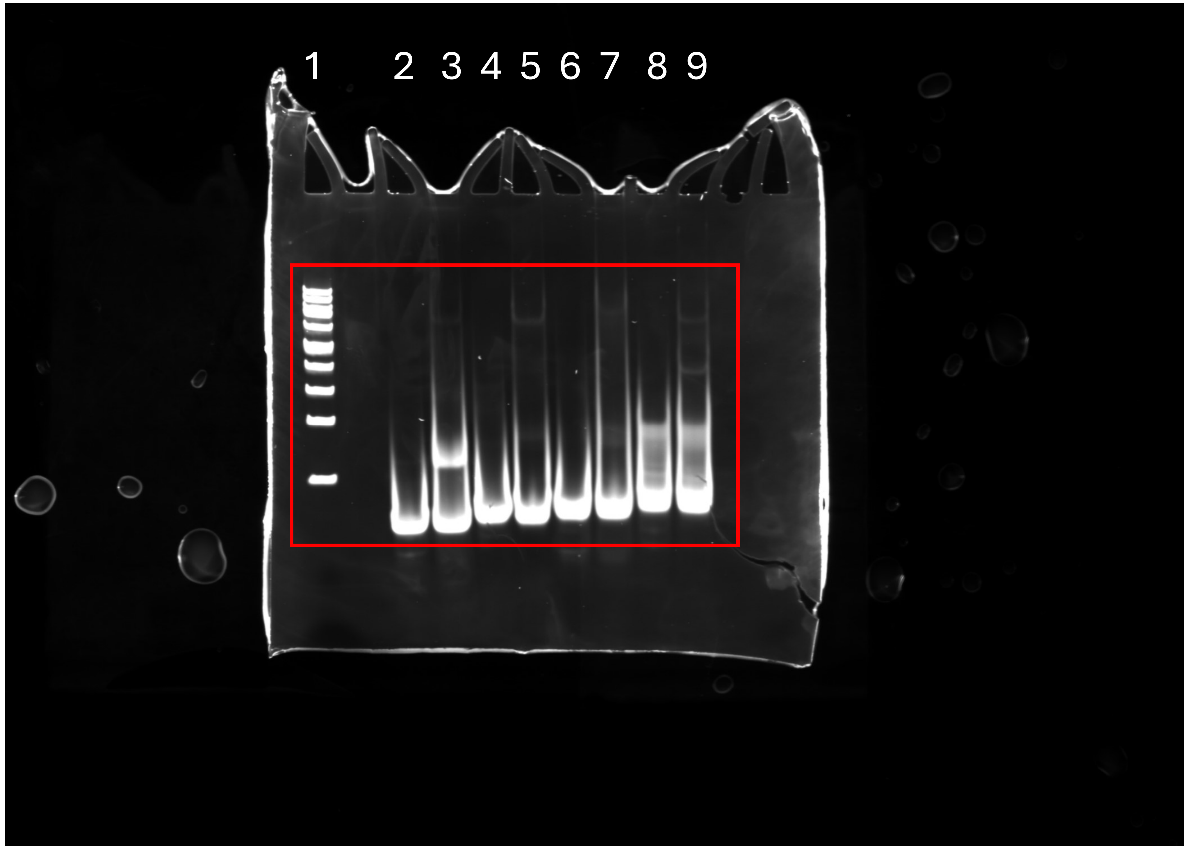

1 100bp ladder

2 rpmB2, unmodified
3 rpmB2, unmodified + Zur protein

4 rpmB2, upper strand ADPr
5 rpmB2, upper strand ADPr + Zur protein

6 rpmB2, lower strand ADPr
7 rpmB2, lower strand ADPr + Zur protein

8 rpmB2, upper and lower strand ADPr
9 rpmB2, upper and lower strand ADPr + Zur protein
