# Supplementary material for: Control of replication and gene expression by ADP-ribosylation of DNA in Mycobacterium tuberculosis
Source: EMBO J. 2025 May 8;44(12):3468–91. doi: 10.1038/s44318-025-00451-y (PMC12170906; doi:10.1038/s44318-025-00451-y)
Supplement: Supplementary file 12 — Source data Fig. 4 [file 44318_2025_451_MOESM12_ESM.zip › Figure 4/4C/rpmB2 cropping.pptx]

## Slide 1
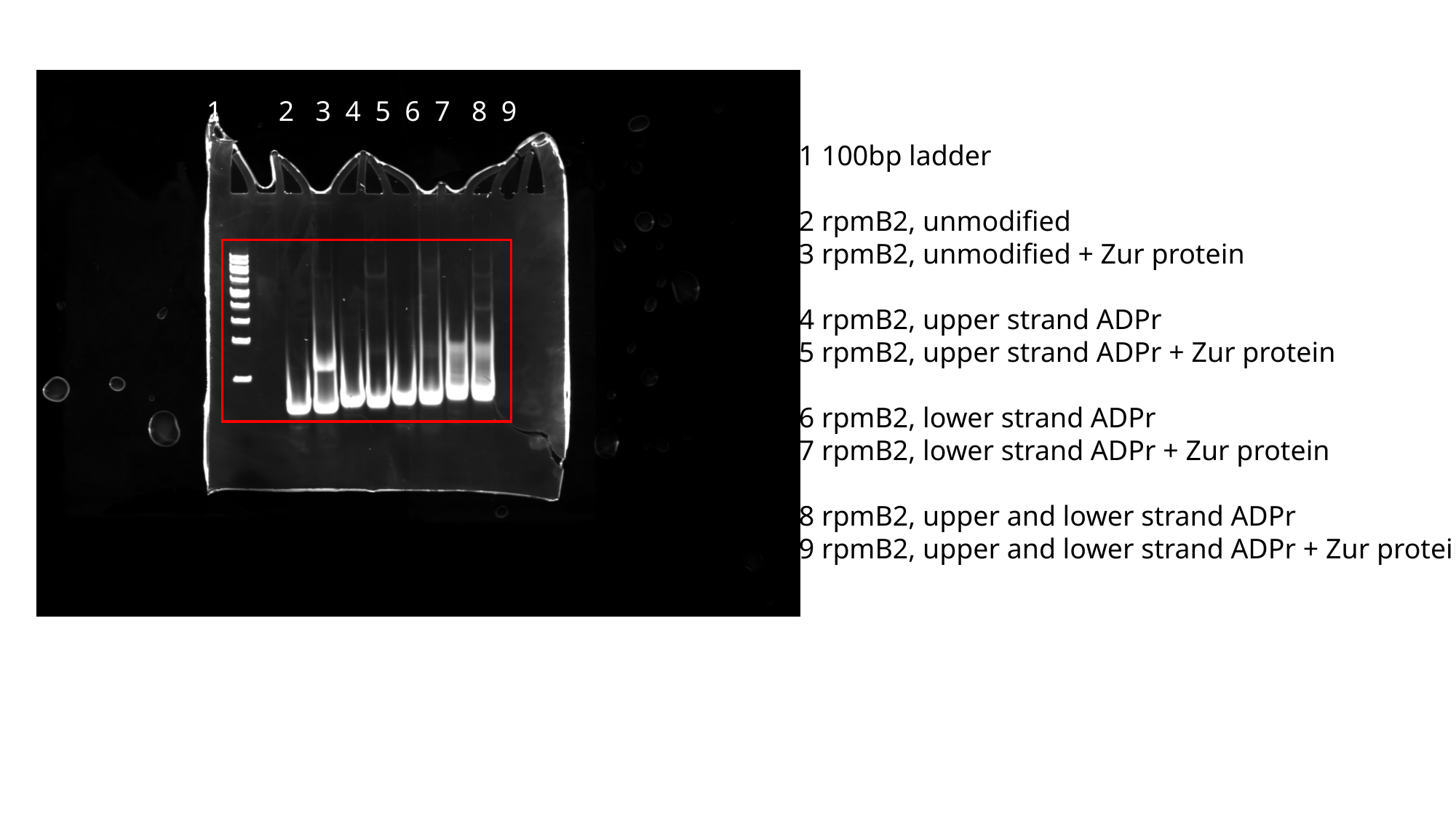

1 2 3 4 5 6 7 8 9
1 100bp ladder
2 rpmB2, unmodified
3 rpmB2, unmodified + Zur protein
4 rpmB2, upper strand ADPr
5 rpmB2, upper strand ADPr + Zur protein
6 rpmB2, lower strand ADPr
7 rpmB2, lower strand ADPr + Zur protein
8 rpmB2, upper and lower strand ADPr
9 rpmB2, upper and lower strand ADPr + Zur protein
