# Supplementary material for: Control of replication and gene expression by ADP-ribosylation of DNA in Mycobacterium tuberculosis
Source: EMBO J. 2025 May 8;44(12):3468–91. doi: 10.1038/s44318-025-00451-y (PMC12170906; doi:10.1038/s44318-025-00451-y)
Supplement: Supplementary file 15 — Expanded View Figures [file 44318_2025_451_MOESM15_ESM.pdf]

Expanded View Figures

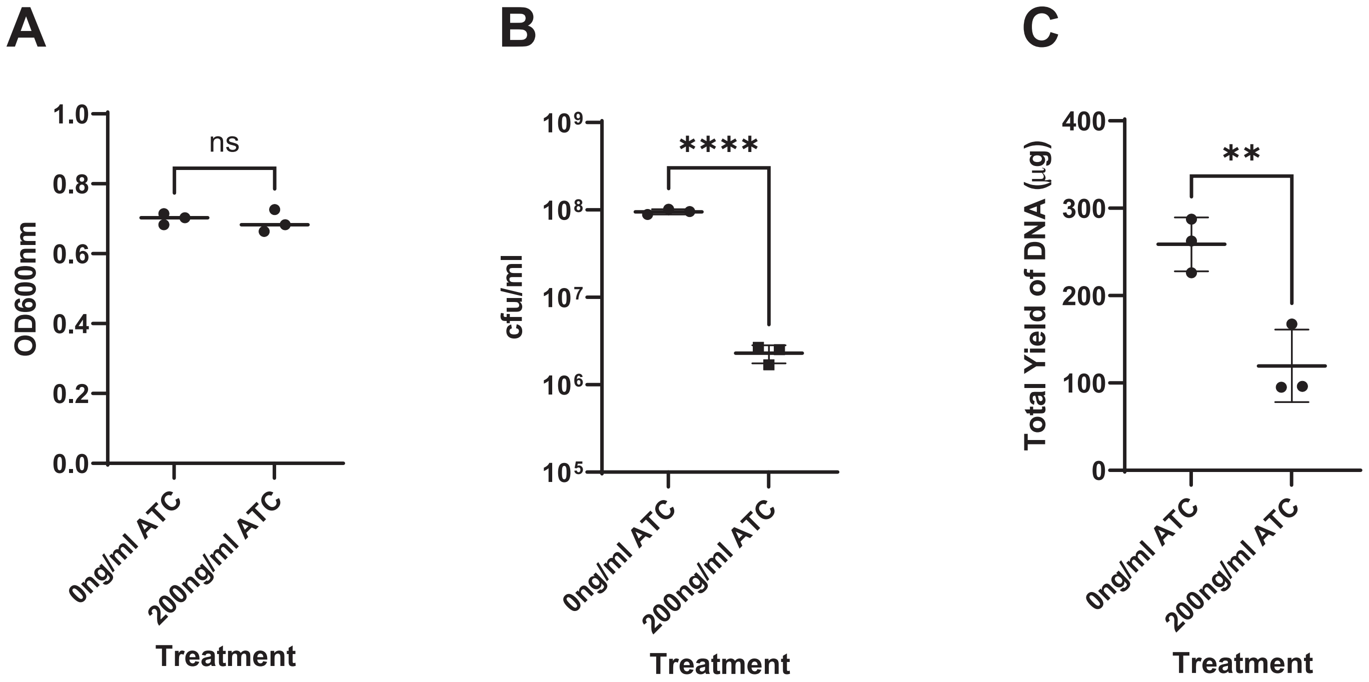

Figure EV1. ***In cellulo* ADP-ribosylation of gDNA inhibits DNA synthesis.**

In cellulo ADP-ribosylation of gDNA in *M. bovis* BCG was induced in late log phase by CRISPRi of the antitoxin *darG* with 200 ng/ml ATC (or 0 ng/ml ATC uninduced control). After 24 h, the cultures were diluted to stimulate replication, and the ATC concentration replenished as indicated. After 3 days, optical density (OD_{600} nm; **A**), and colony forming units (cfu; **B**)) were assessed. Genomic DNA (**C**) was isolated from the cultures by phenol:chloroform:isoamyl extraction and quantified as detailed in "Methods" section. Data are mean +/− SD for $n = 3$ replicate cultures; n.s = not significant, $^{**}P = 0.0096$. $^{****}P = 0.00002$ by unpaired two-tailed T test. Source data are available online for this figure.

 © The Author(s)

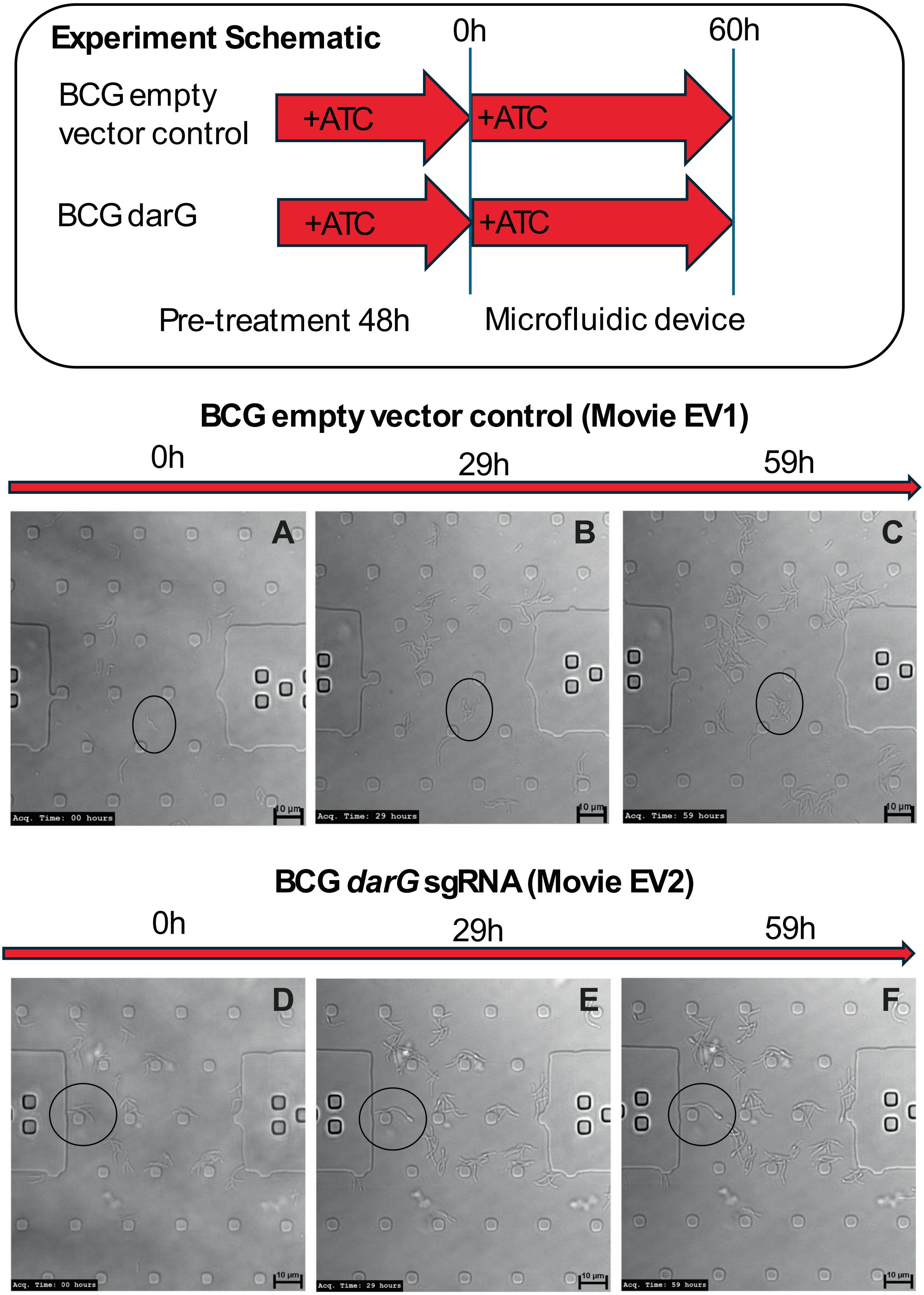
Experiment Schematic
0h
60h
BCG empty vector control
+ATC
+ATC
BCG darG
+ATC
+ATC
Pre-treatment 48h
Microfluidic device
BCG empty vector control (Movie EV1)
0h
29h
59h
A
B
C
Acq. Time: 00 hours
Acq. Time: 29 hours
Acq. Time: 59 hours
10 µm
BCG darG sgRNA (Movie EV2)
0h
29h
59h
D
E
F
Acq. Time: 00 hours
Acq. Time: 29 hours
Acq. Time: 59 hours
10 µm

◀ **Figure EV2. Time-lapse stills from Movies EV1 and EV2.**

An experiment schematic is included in the panel. BCG darG-sgRNA and BCG carrying an empty vector were pretreated for 48 h with 200 ng/ml ATC, then loaded into a microfluidic device. The bacteria were imaged every hour for 59 h, with continued ATC treatment. BCG-vector control bacilli divide over 59 h (**A–C**), whereas BCG darG-sgRNA stop dividing and elongate (**D–F**). An example is circled in each treatment. Source data are available online for this figure.

 © The Author(s)

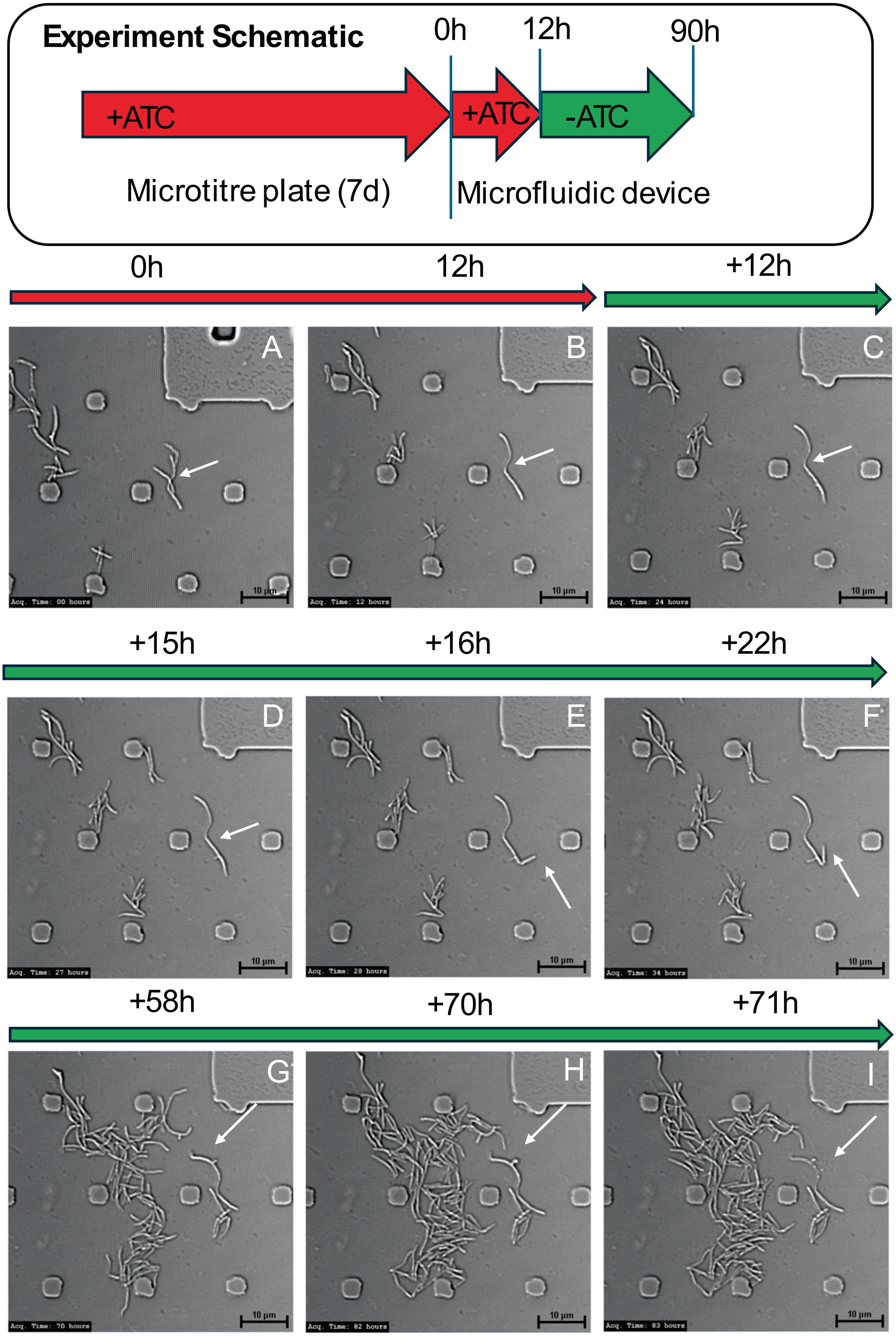
Experiment Schematic
0h
12h
90h
+ATC
+ATC
-ATC
Microtitre plate (7d)
Microfluidic device
0h
12h
+12h
A
B
C
+15h
+16h
+22h
D
E
F
+58h
+70h
+71h
G
H
I

◀ **Figure EV3. Time-lapse stills from Fig. 1D and Movie EV3.**

An experiment schematic is included in the panel. BCG darG-sgRNA were minimally inhibited with ATC in a microtitre plate for 7 days, and loaded into a microfluidic device (**A**). After 12 h, ATC was washed out of the device (**B**). The bacterium marked with a white arrow was followed over time (**C**). After 16 h in fresh medium, division occurs at one pole (**D**–**F**). After 58 in fresh medium, branching occurs at the other pole (**G**) but is followed by swelling (**H**) and necrosis (**I**).

 © The Author(s)

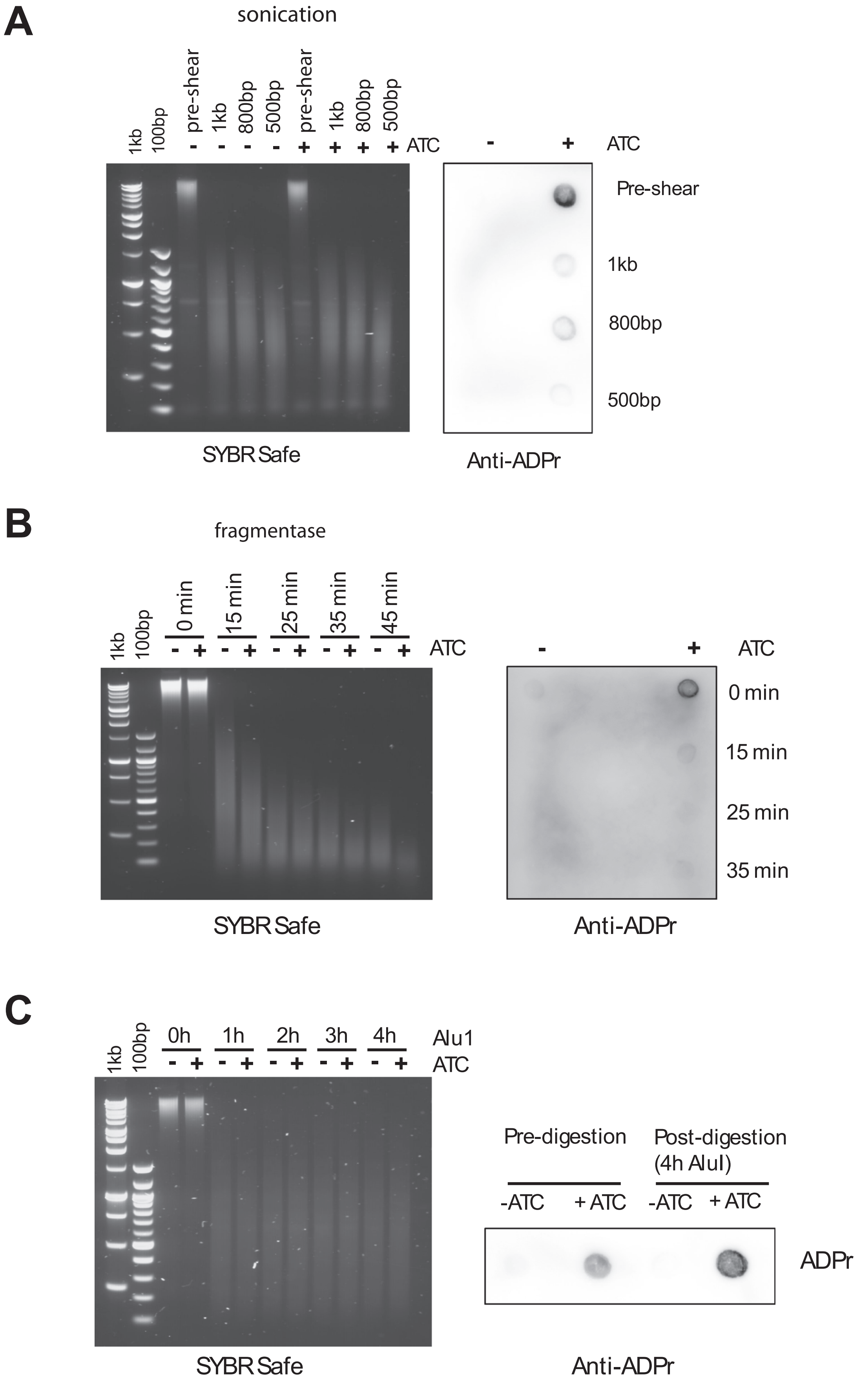
A
sonication
1kb
100bp
pre-shear
1kb
800bp
500bp
pre-shear
1kb
800bp
500bp
- - - - + + + + ATC
- + ATC
Pre-shear
1kb
800bp
500bp
SYBR Safe
Anti-ADPr
B
fragmentase
1kb
100bp
0 min
15 min
25 min
35 min
45 min
- + - + - + - + - + ATC
- + ATC
0 min
15 min
25 min
35 min
SYBR Safe
Anti-ADPr
C
1kb
100bp
0h
1h
2h
3h
4h
Alu1
- + - + - + - + - + ATC
Pre-digestion
Post-digestion (4h AluI)
-ATC
+ ATC
-ATC
+ ATC
ADPr
SYBR Safe
Anti-ADPr

Figure EV4. Survival of the E6F6A ADPr epitope after gDNA fragmentation.

In cellulo ADP-ribosylation of gDNA was induced in BCG by CRISPRi knockdown of the antitoxin *darG* with 200 ng/ml ATC (or 0 ng/ml ATC control) for 48 h. gDNA was extracted and fragmented by (**A**) sonication (S220 Focussed Ultrasonicator, Covaris); (**B**) NEBNext Fragmentase Enzymes (FR); (**C**) AluI digestion. Fragmentation was analysed by agarose gel electrophoresis (left panels). Equal quantities of DNA were bound to a Zetaprobe membrane and epitope stability assessed by dot-blot using the E6F6A antibody against ADP-ribose. The ADPr-Seq technique requires the generation of gDNA fragments that are affinity purified using the anti-ADPr antibody E6F6A, which are then sequenced by NGS. This approach required the ADPr epitope to remain on the DNA following fragmentation. We tried three approaches to generating gDNA fragments. Typically sonication is used to generate 500 bp fragments for NGS studies, however this led to the loss of the ADPr epitope (**A**). Random fragmentation using NEBNext dsDNA Fragmentase also led to loss of the ADPr epitope, and unequal fragment sizes were generated between control gDNA (-ATC) and ADPr-gDNA (+ATC) (**B**). Digestion with AluI maintained the epitope post fragmentation, and resulted in a similar fragmentation pattern in control gDNA (-ATC) and ADPr-gDNA (+ATC) (**C**).

 © The Author(s)

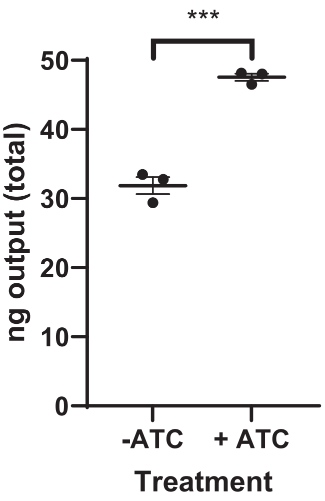

Figure EV5. Yield of DNA fragments recovered by immunoprecipitation with anti-ADPr antibody.

DNA from *darG*-silenced (+ATC) and uninduced control BCG (-ATC) was fragmented with AluI and immunoprecipitated using the E6F6A anti-ADPr antibody. The yield of DNA from each precipitation was measured using the dsDNA Quantifluor system (Promega). Error bars are mean +/− SEM, ****P* = 0.0003 by unpaired T test.

© The Author(s)

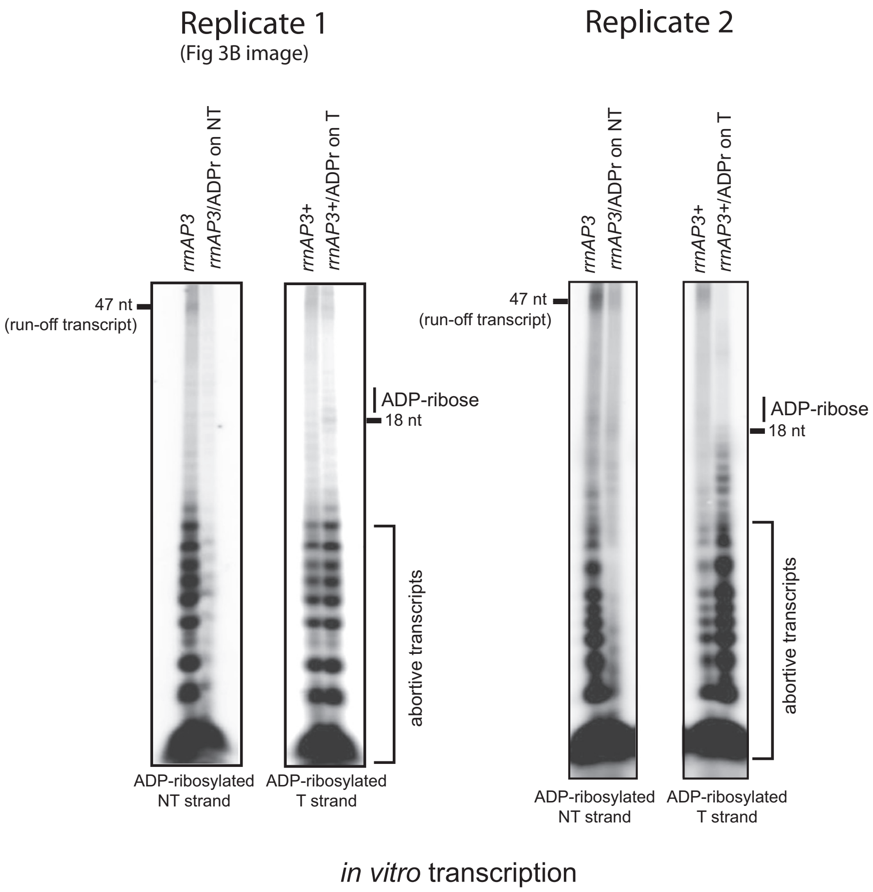

Figure EV6. Replicate in vitro transcription assay showing effects of ADP-ribosylation of ssDNA on transcription by *M. tuberculosis* RNA polymerase.

IVT was performed with the *M. tuberculosis* RNAP holoenzyme on *rrnAP3/*ADPr template (ADP-ribose on non-template NT strand) and *rrnAP3*+/ADPr template (ADP-ribose on template T strand) alongside substrates carrying no ADP-ribose modifications. This showed that ADP-ribosylation of the NT strand of *rrnAP3* inhibited the formation of both abortive transcripts as well as run-off transcripts, whereas ADP-ribosylation on the template strand of *rrnAP3*+ did not inhibit transcription although there was some indication for formation of stalled elongation complex near the introduced ADP-ribosylation site. For direct comparison, Fig. 3B image (replicate 1) is shown alongside replicate 2.

 © The Author(s)
